# Identification of allergens for food-dependent exercise-induced anaphylaxis to shrimp

**DOI:** 10.1038/s41598-021-84752-2

**Published:** 2021-03-08

**Authors:** Shiori Akimoto, Tomoharu Yokooji, Ryohei Ogino, Yuko Chinuki, Takanori Taogoshi, Atsuko Adachi, Eishin Morita, Hiroaki Matsuo

**Affiliations:** 1 Department of Pharmaceutical Services, Graduate School of Biomedical and Health Sciences, Hiroshima University, 1-2-3 Kasumi, Minami-ku, Hiroshima, 734-8553 Japan; 2 Department of Frontier Science for Pharmacotherapy, Graduate School of Biomedical and Health Sciences, Hiroshima University, Hiroshima, Japan; 3 Department of Dermatology, Faculty of Medicine, Shimane University, Izumo, Japan; 4 Department of Dermatology, Hyogo Prefectural Kakogawa Medical Center, Kakogawa, Japan

**Keywords:** Molecular medicine, Diagnostic markers

## Abstract

Shrimp is a causative food that elicits food-dependent exercise-induced anaphylaxis (FDEIA). In this study, we sought to identify IgE-binding allergens in patients with shrimp-FDEIA. Sera were obtained from eight patients with shrimp-FDEIA and two healthy control subjects. Proteins were extracted from four shrimp species by homogenization in Tris buffer. Immunoblot analysis revealed that IgE from patient sera bound strongly to a 70-kDa and a 43-kDa protein in a preparation of Tris-soluble extracts from *Litopenaeus vannamei*. Mass spectrometry identified the 70-kDa and 43-kDa proteins as a P75 homologue and fructose 1,6-bisphosphate aldolase (FBPA), respectively. To confirm that the putative shrimp allergens were specifically recognized by serum IgE from shrimp-FDEIA patients, the two proteins were purified by ammonium sulfate precipitation followed by reversed-phase HPLC and/or anion-exchange hydrophobic interaction chromatography and then subjected to immunoblot analysis. Purified P75 homologue and FBPA were positively bound by serum IgE from one and three, respectively, of the eight patients with shrimp-FDEIA, but not by sera from control subjects. Thus, P75 homologue and FBPA are identified as IgE-binding allergens for shrimp-FDEIA. These findings could be useful for the development of diagnostic tools and desensitization therapy for shrimp-FDEIA patients.

## Introduction

Crustaceans are common causes of immediate-type food allergy and food-dependent exercise-induced anaphylaxis (FDEIA). In Japan, the prevalence of immediate-type food allergy to crustaceans is reported to be 3.4%, predominantly affecting adults^1^. In addition, crustaceans are the second most common cause of FDEIA in Japan after wheat^2^. Among the allergies to crustaceans, shrimp allergy is particularly well studied. The major allergens for immediate-type shrimp allergy are tropomyosin (molecular weight [MW] 37 kDa), arginine kinase (40 kDa), sarcoplasmic calcium-binding protein (20 kDa), and myosin light chain (18–20 kDa). These proteins have been identified as allergens in various species of edible shrimp, such as *Litopenaeus vannamei* (Pacific white shrimp), *Penaeus monodon* (black tiger shrimp), and *Crangon crangon* (North Sea shrimp)^3^. Troponin C (20 kDa), triosephosphate isomerase (28 kDa), and hemocyanin (~ 75 kDa) have been identified as minor shrimp allergens, although this finding has not yet been confirmed for patients with allergies elicited by the main species of edible shrimp, including *L. vannamei* and *P. monodon*^3,4^.

In patients with wheat allergy, the major causative allergens differ depending on the clinical subtype of wheat allergy^3,5–11^. Several water/salt-soluble proteins, including profilin, α-amylase inhibitors, and non-specific lipid transfer protein, have been identified as the major allergens in patients with baker’s asthma, immediate-type wheat allergy, and wheat contact urticaria. In contrast, several water/salt-insoluble proteins, such as gliadin and glutenin, are the major allergens for FDEIA to wheat^3,5–11^. These findings raise the possibility that the major causative allergens may also differ between shrimp-induced immediate-type allergy and shrimp-FDEIA. Previously, we have detected tropomyosin-specific IgE in only 48% (13 out of 27) of the patients with shrimp allergy, indicating that causative shrimp allergens are variable among patients^12^. In addition, we identified a soluble 43-kDa protein from *L. vannamei* and *P. monodon* as an allergen for a patient with shrimp-FDEIA^13^. However, additional major allergens have yet to be determined.

The most reliable treatments for FDEIA are strict elimination of the causative foods and/or exercise limitation after meals. Because these restrictions can deleteriously affect the patient’s quality of life, a precise diagnosis of FDEIA is extremely important to avoid unnecessary elimination diet or exercise-limitation upon doubtful diagnosis. A provocation test, in which subjects exercise after ingestion of the suspected food, is the standard method of diagnosing FDEIA and identifying the specific cause. However, this test is not always safe because subjects could develop severe anaphylaxis during the test. On the other hand, provocation tests with insufficient exercise or insufficient amount of causative foods result in false negative diagnosis. Therefore, there is an unmet need for an accurate and safe in vitro method to diagnose FDEIA.

In clinical settings, levels of serum IgE specific to suspected food proteins are usually measured using the CAP-fluorescent enzyme-immunoassay (CAP-FEIA) system (ImmunoCAP, Thermo Fisher Scientific, Uppsala, Sweden). However, these tests have low sensitivities and specificities and are thus not ideal for the diagnosis of food allergies, including FDEIA^14,15^. Thalayasingam et al. reported that measurement of shrimp-specific IgE by ImmunoCAP had a sensitivity of 62% and a specificity of 50% for detecting shrimp allergy^14^. Similarly, we reported a sensitivity and specificity of 48% and 44%, respectively, for wheat-FDEIA diagnosis by ImmunoCAP measurement of wheat-specific IgE^15^. These low sensitivities and specificities may be due to the heterogeneity of allergenic proteins used in these tests. To overcome this problem, purified native or recombinant allergens, also known as component-resolved diagnostics, is increasingly used for food allergy diagnosis. Using this method, we identified ω5-gliadin and high MW (HMW)-glutenin as two of the major allergens for wheat-FDEIA^16,17^. Furthermore, we reported that measurement of serum IgE levels specific for purified ω5-gliadin was more sensitive and specific for wheat-FDEIA diagnosis compared with measurement of IgE specific for crude wheat or gluten^15^. Takahashi et al. reported that quantification of serum IgE specific for a combination of ω5-gliadin and HMW-glutenin had a sensitivity and specificity of 93.8 and 92.9%, respectively, for wheat-FDEIA diagnosis^18^.

These reports clearly suggest that characterization of causative allergens and measurement of allergen-specific IgE could improve the accuracy of FDEIA diagnosis. In the present study, we sought to identify the causative allergens in patients with shrimp-FDEIA using a combination of chromatographic purification and proteomics analysis.

## Results

### Immunoblot analysis of shrimp proteins

**Figure 1:**
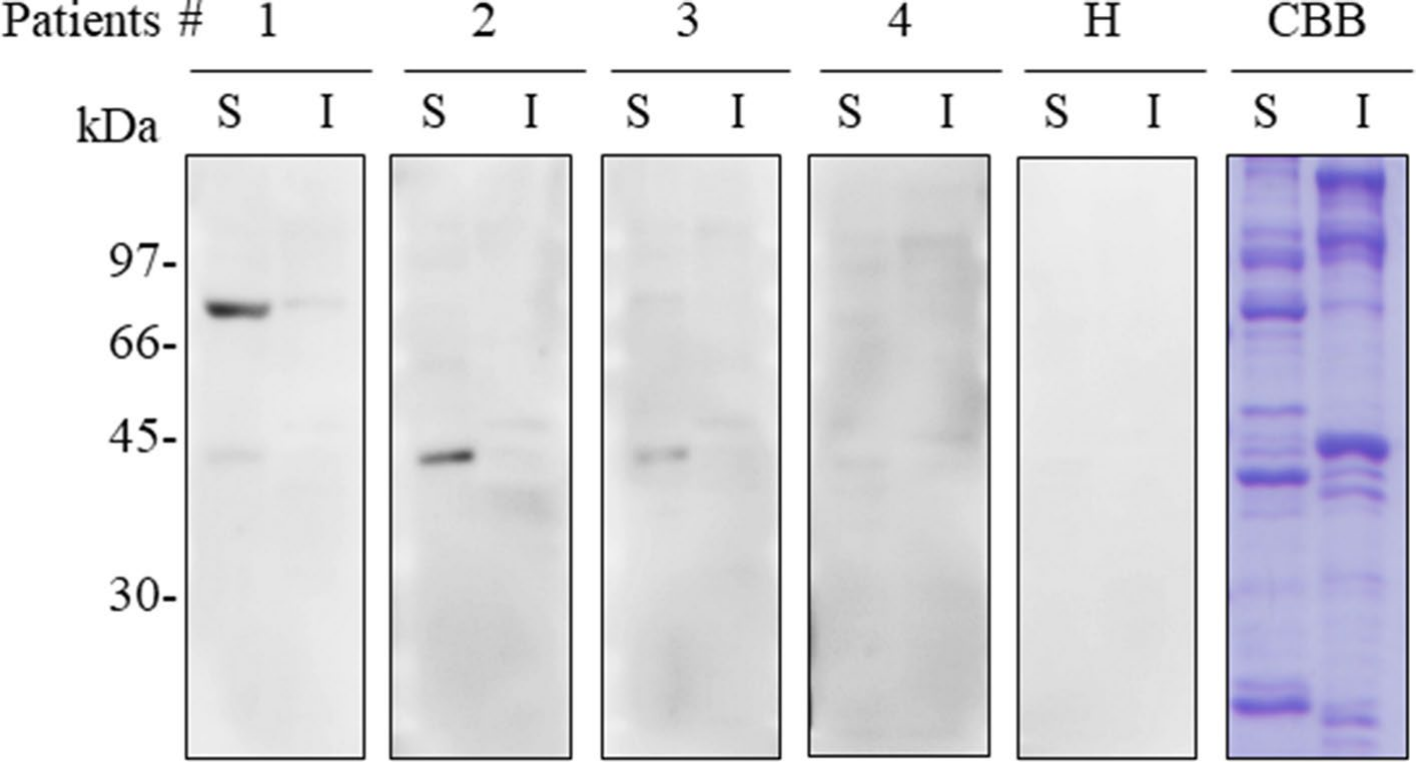
Immunoblot analysis of Tris-soluble and Tris-insoluble shrimp proteins from *L. vannamei*. Samples of the Tris-soluble (S) and Tris-insoluble (I) fractions (10 μg protein) were separated by SDS-PAGE and either stained with CBB or immunoblotted using a 1:10 dilution of sera from shrimp-FDEIA patients nos. 1–4 or a healthy subject (H). Full-length blots and a gel are presented in Supplementary Figure S1.

To investigate potential causative allergens in shrimp-FDEIA, we isolated Tris-soluble and Tris-insoluble shrimp proteins and performed immunoblotting with sera from four patients with shrimp-FDEIA (patient nos. 1–4; Fig. 1). The blots were probed with an IgE-specific secondary antibody to ensure that only IgE binding was detected. Sera from three patients (patient nos. 1–3) reacted with a 43-kDa Tris-soluble protein, and serum from patient no. 1 also reacted with a 70-kDa Tris-soluble protein. However, no binding to Tris-insoluble proteins were observed for any of the patient sera. Serum from a healthy subject did not react with any Tris-soluble or Tris-insoluble shrimp proteins. These results suggested that the IgE-binding 70-kDa and/or 43-kDa Tris-soluble proteins were potential allergens for patients with shrimp-FDEIA.

### IgE cross-reactivity of allergens from four shrimp species

**Figure 2:**
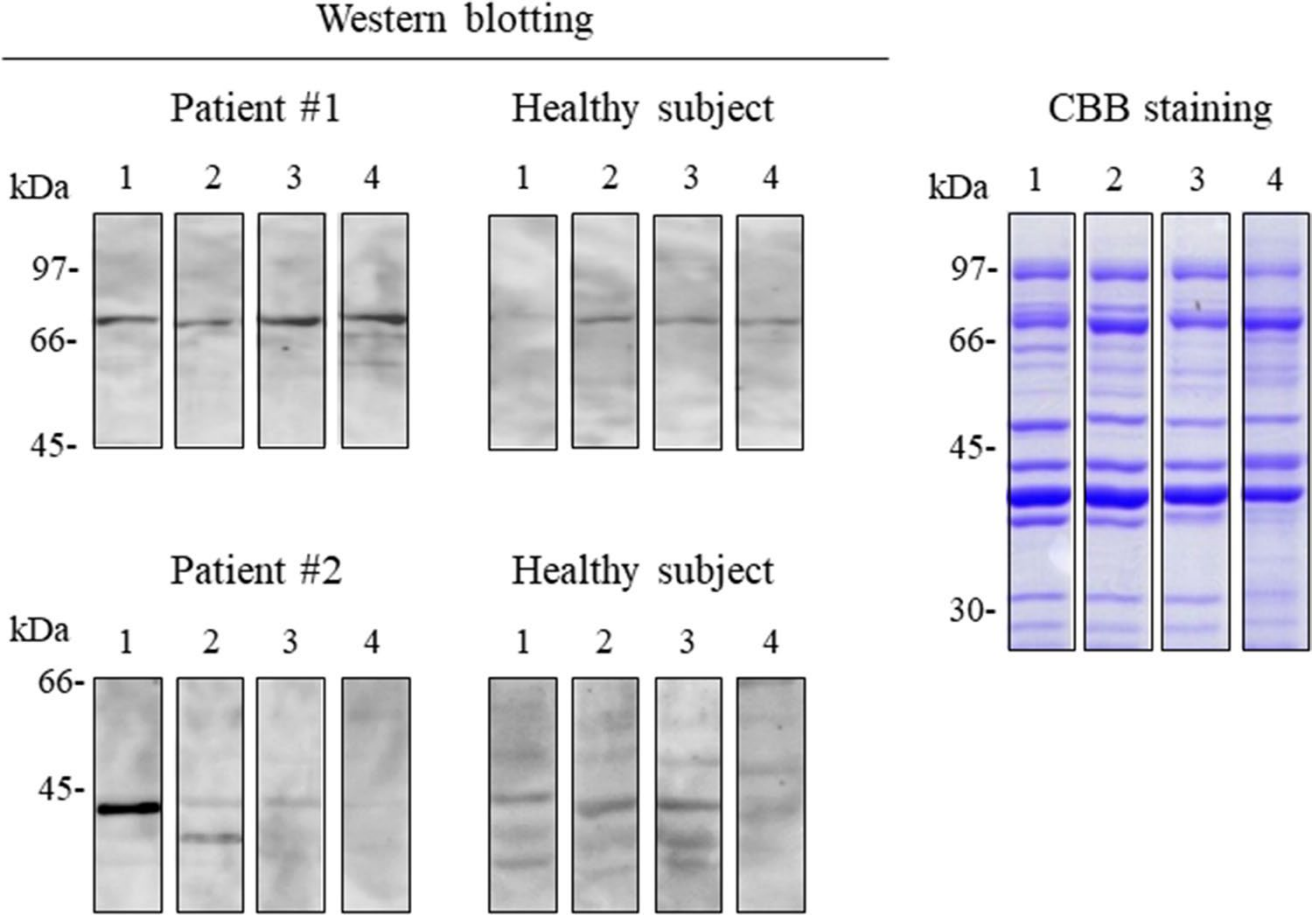
Immunoblot analysis of Tris-soluble shrimp proteins from two shrimp and two prawn species. Samples of the Tris-soluble fraction (10 μg) from *L. vannamei, P. monodon*, *M. japonicus,* and *F. chinensis* (lanes 1, 2, 3, and 4, respectively) were separated by SDS-PAGE and stained with CBB or immunoblotted with 1:10 dilutions of sera from two shrimp-FDEIA patients (patient no. 1 for 70-kDa protein and no. 2 for 43-kDa protein). Each blot was cropped from the same membrane and rearranged. Full-length blots and a gel are presented in Supplementary Figure S2.

To determine whether IgE in sera from patients nos. 1 and 2 cross-reacted with the same allergens isolated from other shrimps, we performed immunoblot analysis of Tris-soluble proteins extracted from *L. vannamei*, *P. monodon*, *Marsupenaeus japonicus*, and *Fenneropenaeus chinensis,* all of which are commonly consumed in Japan. CBB staining of the Tris-soluble proteins separated by SDS-PAGE revealed virtually the same protein band patterns for all four shrimp species (Fig. 2). Interestingly, serum IgE reactivity differed for proteins from the four shrimp species. Whereas serum from patient no. 1 bound to a 70-kDa protein from all four species, only the 70-kDa protein from *L. vannamei* was not recognized by sera from the healthy subjects, indicating that serum reactivity with the 70-kDa band isolated from *L. vannamei* was most specifically compared to that from *P. monodon*, *M. japonicus,* and *F. chinensis*. In addition, patient no. 2 serum showed strong IgE reactivity to a 43-kDa protein from *L. vannamei,* but a protein of the same approximate MW from the other three shrimp species was bound only weakly or not at all (Fig. 2). Thus, we selected the Tris-soluble proteins from *L. vannamei* for further investigation.

### Identification of the 70-kDa and 43-kDa Tris-soluble proteins from L. vannamei

**Figure 3:**
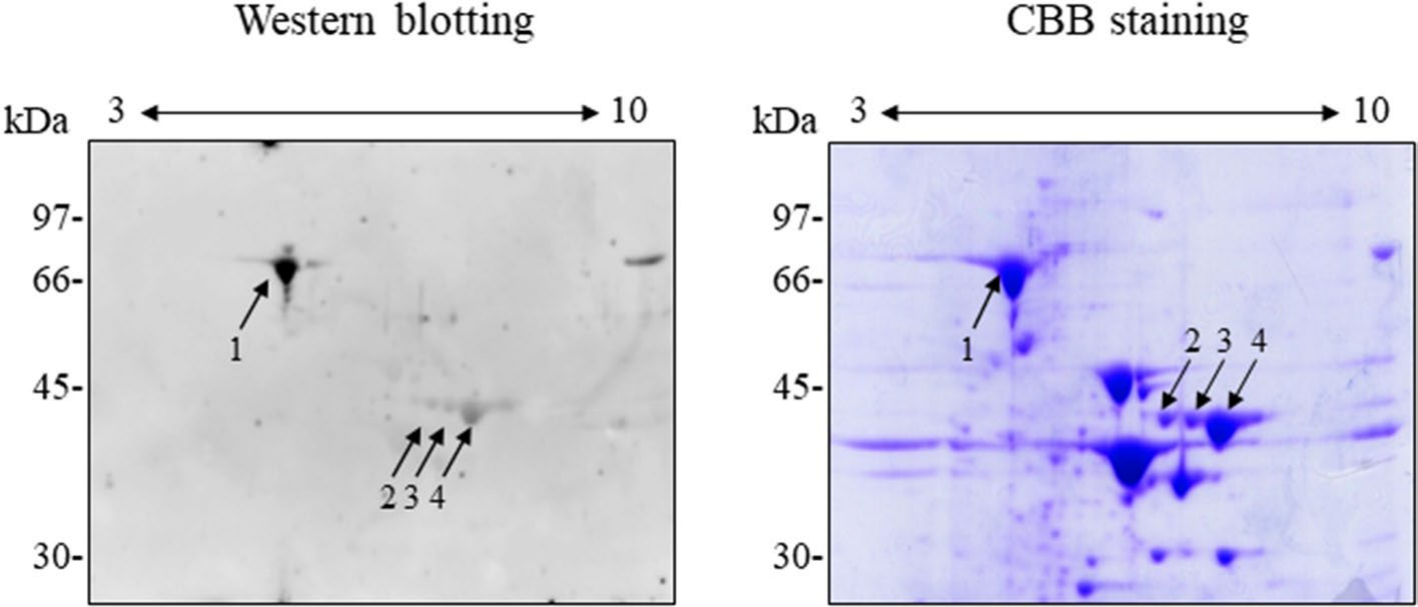
2D-PAGE and immunoblot analysis of two putative allergens from *L. vannamei*. Samples of the Tris-soluble protein fraction (100 μg) were separated by 2D-PAGE and either stained with CBB or immunoblotted with a 1:10 dilution of serum from shrimp-FDEIA patient no. 1. Arrows indicate the dominant IgE-binding proteins. A full-length blot and a gel are presented in Supplementary Figure S3.

To identify the two IgE-binding Tris-soluble proteins, we performed 2D-PAGE, extracted the relevant spots, and subjected them to matrix-assisted laser desorption/ionization time of flight mass spectrometry (MALDI-TOF MS). As shown in Fig. 3, one spot was detected at 70 kDa with a p*I* of 6 (spot 1) and three spots were detected at 43 kDa with p*I*s of 7.2 (spot 2), 7.6 (spot 3), and 7.9 (spot 4). After MALDI-TOF MS, the mass lists were searched using MASCOT software. The amino acid sequences of the putative proteins were deduced from the *L. vannamei* cDNA sequence (accession no. FE099401) and a homology search was performed using basic local alignment search tool (BLAST)-N^19^. Spot 1 was found to contain a 230-amino acid sequence with 100% identity to amino acids 249–478 of Nesprin-1 like protein (accession no. ROT70123). The sequence of amino acids 106–196 of Nesprin-1-like protein is 80.0% identical to a partial sequence of fast muscle P75-like protein from American lobster (*Homarus americanus*, accession no. AY302591)^20^. Therefore, spot 1 was considered to be a shrimp homologue of fast muscle P75 protein. Using the same approach, spots 2, 3, and 4 were all identified as components of fructose 1,6-bisphosphate aldolase (FBPA, accession no. MK840979).

### Chromatographic purification of P75 homologue and FBPA from L. vannamei

To enable confirmation of IgE binding from shrimp-FDEIA patients to the putative allergens P75 homologue and FBPA, we purified the *L. vannamei* native proteins from the Tris-soluble shrimp protein extract by ammonium sulfate precipitation followed by chromatography.

**Figure 4:**
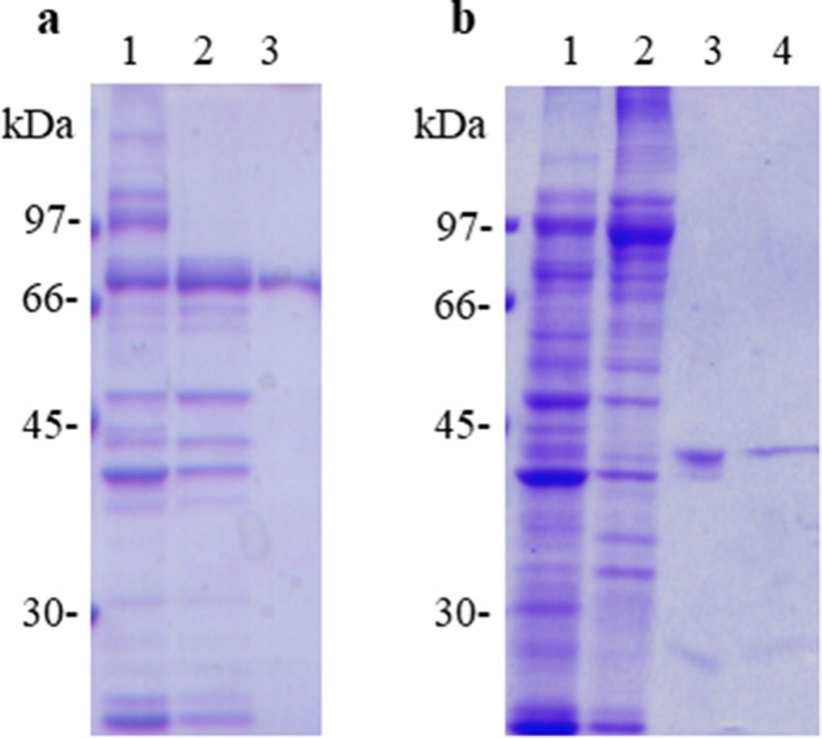
Purification of P75 homologue and FBPA proteins from *L. vannamei*. (**a**) CBB staining of a 70-kDa protein in the whole Tris-soluble fraction (lane 1), the 40–60% ammonium sulfate precipitate (lane 2), and reversed-phase HPLC fraction (lane 3). (**b**) CBB staining of a 43-kDa protein in the whole Tris-soluble fraction (lane 1), 20–40% ammonium sulfate precipitate (lane 2), anion-exchange chromatography fraction (lane 3), and hydrophobic interaction chromatography fraction (lane 4). Full-length gels are presented in Supplementary Figure S4.

To isolate the P75 homologue, the 40–60% ammonium sulfate precipitate was fractionated by phase high performance liquid chromatography (HPLC) using a linear gradient of 30–70% acetonitrile in 10 mM phosphate buffer (pH 7.0). Protein-containing fractions were analyzed by SDS-PAGE and CBB staining (Fig. 4) or immunoblot analysis (data not shown), which revealed a single band corresponding to the P75 homologue eluted in fractions containing ~ 45–65% acetonitrile in 10 mM phosphate buffer.

For FBPA isolation, the 20–40% ammonium sulfate precipitate was fractionated by HPLC using an anion-exchange column with a linear gradient of 0−50% Tris–HCl (pH 8.0) containing 1 M NaCl. Fractions (~ 0.15–0.3 M NaCl in Tris–HCl, pH 8.0) contained several bands by SDS-PAGE and immunoblot analysis with serum from patient no. 1. These fractions were pooled and subjected to hydrophobic interaction chromatography. CBB staining (Fig. 4) and immunoblot analysis (data not shown) of the resulting fractions showed a single band corresponding to FBPA in fractions containing 0.75–0.5 M ammonium sulfate in Tris–HCl (pH 8.0). Collectively, these data indicated that native P75 homologue and FBPA proteins were successfully isolated with high purity from a Tris-soluble extract of *L. vannamei*.

### Immunoblot analysis of native P75 homologue and FBPA

**Figure 5:**
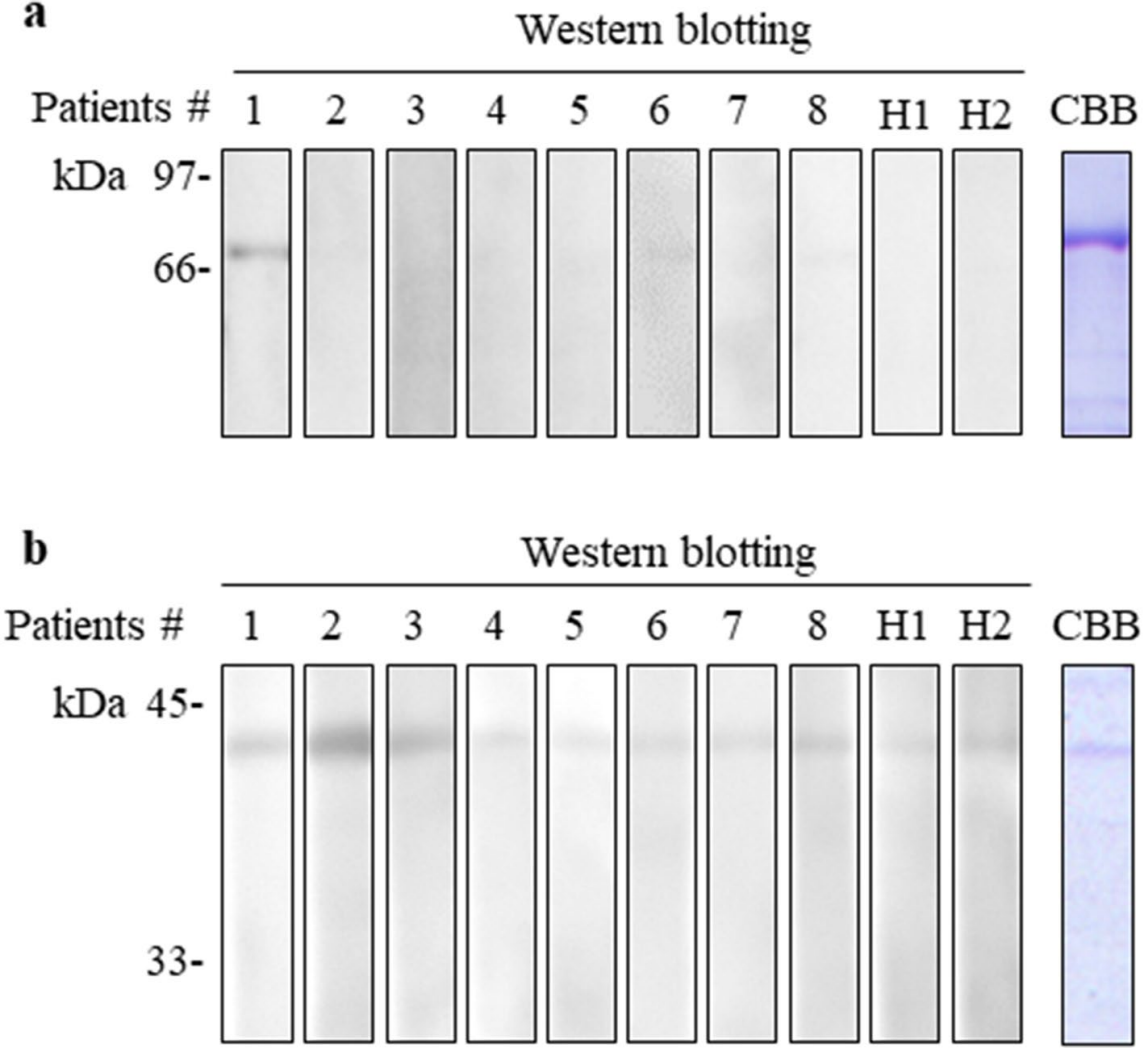
Immunoblot analysis of P75 homologue and FBPA proteins isolated from *L. vannamei.* Samples of purified native P75 homologue (**a**, 0.24 µg) and FBPA (**b**, 1.2 µg) were subjected to SDS-PAGE and either stained with CBB or immunoblotted with 1:10 dilutions of sera from shrimp-FDEIA patient nos. 1–8 and healthy subjects (H1 and H2). Full-length blots and gels are presented in Supplementary Figure S5.

Finally, we determined whether the purified *L. vannamei* P75 homologue and FBPA proteins were specifically recognized by serum IgE from the eight patients with shrimp-FDEIA. We found that the P75 homologue was recognized by serum from patient no. 1 but not by sera from the remaining seven patients or two healthy subjects (Fig. 5). Purified FBPA was recognized by sera from patient nos. 1, 2, and 3, but not by sera from patient nos. 4–8 or from the healthy subjects. Notably, IgE in sera from 13 patients with immediate-type shrimp allergy did not react with either of the purified proteins (data not shown). Thus, P75 homologue and FBPA from *L. vannamei* may be allergens specifically for shrimp-FDEIA.

## Discussion

Although the ImmunoCAP test is often used to measure serum levels of IgE specific for suspected food allergens, its sensitivity and specificity are low^14,15^, possibly because of protein heterogeneity. In the case of suspected shrimp allergy, the ImmunoCAP test employs a mixture of crude extracts from *P. monodon*, *Metapenaeus joyneri*, *Metapenaeopsis barbata,* and *Pandalus borealis*, and has a reported sensitivity and specificity of 62% and 50%, respectively, for the diagnosis of shrimp allergy^14^. A component-resolved diagnostic approach is now being employed to overcome the unsatisfactory sensitivity/specificity levels of food allergy tests that use heterogeneous protein mixtures. In the present study, we identified P75 homologue and FBPA as new potential allergens for shrimp-FDEIA using proteomic analysis. We further confirmed that these proteins purified by chromatography methods specifically reacted with IgE from patients with shrimp-FDEIA. This is the first report that P75 homologue and FBPA are candidates of specific allergens for shrimp-FDEIA.

We initially found that serum IgE from patients with shrimp-FDEIA bound strongly to 70-kDa and 43-kDa Tris-soluble proteins. The MWs of these proteins indicated that they were unlikely to be any of the known shrimp allergens; namely, tropomyosin (37 kDa), arginine kinase (40 kDa), sarcoplasmic calcium-binding protein (20 kDa), myosin light chain (18–20 kDa), troponin C (20 kDa), triosephosphate isomerase (28 kDa), and hemocyanin (~ 75 kDa)^3,4^. Although hemocyanin is a similar size to the 70-kDa protein, it was excluded as a candidate because sera from our patients did not react to any proteins in shrimp hemolymph, which contains hemocyanin (data not shown). In addition, tropomyosin, a major shrimp allergen, was excluded as a candidate because four patients (nos. 1–4) did not react to tropomyosin (Fig. 1) and two of three patients (nos. 6 and 7) showed negative by CAP-FEIA for tropomyosin (Table 1).

**Table 1:** Clinical characteristics of patients with shrimp-FDEIA.

No	Age (years)	Gender	Symptoms	Provocation test	Total IgE (IU/mL)	Specific IgE (Ua/mL)		
						Shrimp	Crab	Tropomyosin
1	17	F	D, U, C	Positive	1,552	1.13	1.13	ND
2	16	M	U, P	Positive	1,210	< 0.35	3.99	ND
3	45	M	H, U, S	Negative	4,468	11.4	12.4	ND
4	13	F	H, U, D	Positive	568	< 0.35	< 0.35	ND
5	18	F	A	Positive	4	< 0.35	< 0.35	ND
6	14	F	U	ND	97	< 0.35	< 0.35	< 0.35
7	24	F	U	ND	61	< 0.35	< 0.35	< 0.35
8	28	M	D, AP, U	ND	848	2.82	ND	2.35

Serum IgE reactivity to shrimp and crab proteins was analyzed by CAP-FEIA. M, male; F, female; ND, not determined. D, dyspnea; U, urticaria; C, cough; P, pruritus; H, headache; S, shock; A, anaphylaxis; AP, abdominal pain.

The results of our MS analysis identified the 70-kDa and 43-kDa proteins as a homologue of fast muscle P75-like protein and FBPA, respectively. P75 was identified as a myofibrillar protein of crustaceans such as *H. americanus* and *Ocypode stimpsoni* as well as myosin heavy chain (MHC), troponin I and troponin T though its function remains unknown^20,21^. Although MHC and troponin I are known allergens for immediate-type shrimp allergy, this has not been reported for P75 or homologues^22,23^. Thus, the P75 homologue identified here is a new candidate allergen for shrimp-FDEIA.

FBPA is an essential glycolytic enzyme that catalyzes the reversible conversion of fructose 1,6-bisphosphate to glyceraldehyde 3-phosphate and dihydroxyacetone phosphate. FBPA from Pacific salmon was the first to be identified as an allergen^24^, after which FBPA from *Forcipomyia taiwana*, Anisakis, fungi, and *Manihot esculenta* were also shown to be allergenic^25–28^. Gamez et al. reported that FBPA from *Solenocera melantho* is a shrimp allergen^29^. However, with the exception of our earlier case report^13^, FBPA has not been reported to be an allergen for shrimp-FDEIA. Thus, our study has identified two new potential allergens for shrimp-FDEIA.

Several case studies have shown that cross-reactivity among crustacean proteins is a frequent cause of allergic symptoms in patients with shrimp allergy. This may be due to the high degree of conservation of amino acid sequences in the epitopes recognized by IgE. For example, Ruethers et al. reported that the amino acid sequence of tropomyosin is 88% to 100% identical among crustaceans^23^. Here, we examined the potential cross-reactivity of P75 homologue and FBPA from four shrimp species. We found that serum IgE from one shrimp-FDEIA patient reacted with P75 homologue isolated from the four shrimp species, suggesting that this protein may be a cross-reactive allergen among crustaceans. In contrast to P75, only FBPA isolated from *L. vannamei* was recognized by serum IgE from one shrimp-FDEIA patient, even though FBPA from *L. vannamei* and *F. chinensis* are 95% identical. It is possible that the lack of cross-reactivity to FBPA is due to the presence of distinct immunodominant IgE epitopes in the two proteins. Further work will be necessary to elucidate the cross-reactivity of P75 homologue and FBPA from different shrimp species and other crustaceans.

Of the eight patients with shrimp-FDEIA whose serum reactivity was examined in the present study, one (12.5%) and three (37.5%) sera recognized P75 and FBPA, respectively. Reactivity to neither protein was detected in sera from healthy subjects or from patients with immediate-type shrimp allergy (data not shown), suggesting that P75 homologue and/or FBPA may be specific allergens for the FDEIA subtype of shrimp allergy. One limitation of this study is the small number of patients investigated; thus, further work will be necessary to confirm our results.

Recently, several studies have achieved some success for desensitization immune therapy for patients with food allergies. However, desensitization therapy using crude extracts from natural foods has often failed due to limited efficacy and potential anaphylactic side effects^30^. Thus, the results of the present study may be useful for the development of safe and effective desensitization therapies for patients with shrimp-FDEIA.

In conclusion, we have identified P75 homologue and FBPA as potential novel allergens for FDEIA to shrimp. Our findings have implications for the development of diagnostic tools and therapeutic strategies for shrimp-FDEIA patients.

## Materials and methods

### Subjects

Sera were collected from eight patients with shrimp-FDEIA, as diagnosed by recurrent episodes of exercise-induced anaphylaxis after shrimp ingestion. Provocation tests were performed with shrimp (*P. monodon*) according to a slight modification of previously described method^31^. This test included exercise, shrimp ingestion, aspirin intake (a co-factor) and a combination of these challenges. None of the patients tested exhibited symptoms in response to either exercise, shrimp ingestion or aspirin intake alone. In contrast, four of five patients exhibited allergic symptoms such as urticaria, pruritus, and/or dyspnea when they ingested shrimp followed by the exercise challenge combined with aspirin intake. Sera from two healthy subjects without food allergy were used as negative controls. The sera were stored at − 80 °C until use. The clinical features of the patients are shown in Table 1. This study was approved by the ethics committee of Hiroshima University (approval No. E-580) in compliance with the Declaration of Helsinki and current legal regulations in Japan. The study was explained and written informed consent was obtained from all patients. Informed consent was obtained from parent/legal guardian for the patients having age less than 18.

### Preparation of shrimp proteins

Samples of shucked *L. vannamei*, *P. monodon*, *F. chinensis*, and *M. japonicus* (10 g each) were homogenized in 15 mL of ice-cold 40 mM Tris–HCl buffer (pH 8.0), and each homogenate was centrifuged at 13,200 g for 20 min at 4 °C. The supernatant was collected and reserved, and the pellet was re-homogenized using the same buffer and centrifugation conditions. The two supernatants were mixed and designated the Tris-soluble protein fraction. The pellet was the mixed with 130 mM Tris–HCl buffer containing 6% (w/v) SDS, 10% (v/v) 2-mercaptoethanol, 20% (v/v) glycerol, and 0.02% (w/v) bromophenol blue (BPB). The sample was boiled for 5 min and centrifuged, and the resulting supernatant was collected and designated the Tris-insoluble protein fraction. Protein concentrations were determined using a protein assay kit (Bio-Rad Laboratories, Hercules, CA, USA).

### Purification of shrimp allergens

Tris-soluble proteins from *L. vannamei* were precipitated with ammonium sulfate at concentrations of 0–20%, 20–40%, 40–60%, and > 60% at 4 °C. Each precipitate was dissolved in 65 mM Tris–HCl buffer containing 3% (w/v) SDS, 5% (v/v) 2-mercaptoethanol, 10% (v/v) glycerol, and 0.01% (w/v) BPB. Immunoblot analysis of the precipitates identified 70 kDa and 43 kDa IgE-binding proteins in the 40–60% and 20–40% ammonium sulfate precipitates, respectively.

To obtain the 70-kDa protein, the 40–60% ammonium sulfate precipitate was resuspended in 40 mM Tris–HCl (pH 7.0) and centrifuged at 13,200 g for 5 min at 4 °C. The supernatant was fractionated by reversed-phase HPLC using a YMC-Pack C_4_ column (10 µm, 30 nm, 100 mm × 4.6 mm; YMC, Kyoto, Japan) with a linear gradient of 30 − 70% acetonitrile in 10 mM phosphate buffer (pH 7.0) over 43 min at a flow rate of 0.5 mL/min. Protein elution was monitored by absorbance at 280 nm. The 70-kDa protein peak fractions were collected, pooled, and evaporated.

To obtain the 43-kDa protein, the 20–40% ammonium sulfate precipitate was suspended in 40 mM Tris–HCl (pH 8.0) and centrifuged at 83,000 g for 10 min at 4 °C. The supernatant was fractionated by HPLC (AKTA FPLC system, GE Healthcare, Little Chalfont, UK) using an anion-exchange column (Resource Q 1 mL, 0.64 × 3 cm, GE Healthcare) with a linear gradient of 0 − 50% mobile phase B (Tris–HCl, pH 8.0, 1 M NaCl) in mobile phase A (Tris–HCl, pH 8.0) over 17.5 min at a flow rate of 2 mL/min. Protein elution was monitored by absorbance at 280 nm. To further purify the 43-kDa protein, the 43-kDa-containing fractions from anion-exchange chromatography were applied to a hydrophobic interaction column (HiTrap Butyl FF 1 mL, 0.7 × 2.5 cm, GE Healthcare) and fractionated by stepwise elution with ammonium sulfate at 1 M to 0 M in 0.25 M intervals.

The final preparations confirmed to contain the 70-kDa and 43-kDa proteins by Coomassie brilliant blue (CBB) staining of SDS-PAGE gels and immunoblot analysis using sera from patients (nos. 1 and 2) with shrimp-FDEIA.

### Immunoblot analysis of shrimp proteins

The Tris-soluble and -insoluble shrimp fractions (10 µg/lane) were separated by 10% SDS-PAGE and either stained with CBB or transferred electrophoretically to a nitrocellulose membrane (BioTrace NT Nitrocellulose Transfer Membranes, PALL, Port Washington, NY, USA). The membrane was blocked with 5% skim milk in Tris-buffered saline (pH 7.6) containing 0.1% Tween-20 (TBS-T) for 2 h and washed three times with TBS-T for 10 min each at room temperature. The membrane was then incubated overnight at 4 °C with 1:10 dilutions of sera from the eight patients and two healthy donors, washed with TBS-T, and incubated with a 1:10 dilution of mouse monoclonal anti-human IgE (ImmunoCAP specific IgE Conjugate 400, Thermo Fisher Scientific) for 1 h at room temperature. The membrane was washed three times with TBS-T for 10 min each and then incubated with a 1:1000 dilution of horseradish peroxidase-conjugated horse anti-mouse IgG (Cell Signaling Technology, Danvers, MA, USA) for 1 h. The membrane was washed again with TBS-T and immunoreactive protein bands were revealed using Western Lightning Ultra detection reagents (PerkinElmer, Waltham, MA, USA) and imaged with a LAS-4000mini image analyzer (GE Healthcare).

### Two-dimensional PAGE (2D-PAGE)

To enrich the allergen proteins, the Tris-soluble protein fraction from *L. vannamei* was precipitated with 10% (v/v) trichloroacetic acid (TCA) at − 20 °C for 1 h and centrifuged. The pellet was suspended in ice-cold acetone, incubated at − 20 °C for 1 h, and the suspension was centrifuged at 15,000 g for 10 min at 4 °C. The supernatant was discarded and the precipitate was dissolved in ReadyPrep Rehydration buffer (Bio-Rad) containing 8 M urea, 2% (w/v) CHAPS, 50 mM dithiothreitol (DTT), 0.2% (w/v) Bio-Lyte 3/10 ampholytes, and 0.001% (w/v) BPB. The samples were then subjected to 2D-PAGE as described by Matsuo et al^7^. The first-dimension electrophoresis was performed using a 7-cm immobilized pH gradient gel (IPG) strip with a linear pH 3–10 gradient (Ready Strip, Bio-Rad). The IPG strip was rehydrated with 125 µL of ReadyPrep Rehydration buffer (Bio-Rad) containing 100 µg of TCA-precipitated Tris-soluble shrimp proteins for 12 h. Isoelectric focusing was carried out using a Protean IEF Cell (Bio-Rad) for 10,000 V-hours at 20 °C. The IPG strip was incubated in equilibration buffer containing 6 M urea, 2% (w/v) SDS, 0.357 M Tris–HCl (pH 8.8), 20% (v/v) glycerol, and 2% (w/v) DTT three times for 10 min each. For the second-dimension, the strip was placed on a 10% SDS-PAGE gel and subjected to electrophoresis. Immunoblot analysis was performed as described above to detect the IgE-binding proteins.

### Mass spectrometry (MS) and identification of IgE-binding shrimp proteins

The IgE-binding shrimp proteins were identified as reported previously^32^. Briefly, Tris-soluble proteins were separated by 2D-PAGE and visualized by CBB staining and immunoblot analysis. The spots corresponding to the 70-kDa and 43-kDa proteins were then excised and subjected to alkylation and digestion with iodoacetamide and trypsin, respectively. The mass spectra of peptides were recorded in the reflectron mode of MALDI-TOF MS (AB SCIEX TOF/TOF 5800 system, Framingham, MA, USA). Data processing was carried out using Data Explorer software ver. 4.10 (Applied Biosystems/MDS Analytical Technologies, Foster City, CA, USA). For the MS/MS ion search, the generated mass lists were searched against the protein data bank of the National Center for Biotechnology Information using the database search engine MASCOT (Matrix Science, London, UK) after excluding peak lists of known contaminants such as keratin and trypsin.

## Supplementary Information

Supplementary Information
