# Supplementary material for: Identification of allergens for food-dependent exercise-induced anaphylaxis to shrimp
Source: Sci Rep. 2021 Mar 8;11:5400. doi: 10.1038/s41598-021-84752-2 (PMC7940642; doi:10.1038/s41598-021-84752-2)
Supplement: Supplementary file 1 — Supplementary Information [file 41598_2021_84752_MOESM1_ESM.pdf]

Identification of allergens for food-dependent exercise-induced anaphylaxis to shrimp

Shiori Akimoto[1], Tomoharu Yokooji[1,2*], Ryohei Ogino[3], Yuko Chinuki[3], Takanori Taogoshi[1], Atsuko Adachi[4], Eishin Morita[3], Hiroaki Matsuo[1]

[1]Department of Pharmaceutical Services, Graduate School of Biomedical and Health Sciences, Hiroshima University, Hiroshima, Japan
[2]Department of Frontier Science for Pharmacotherapy, Graduate School of Biomedical and Health Sciences, Hiroshima University, Hiroshima, Japan
[3]Department of Dermatology, Faculty of Medicine, Shimane University, Izumo, Japan
[4]Department of Dermatology, Hyogo Prefectural Kakogawa Medical Center, Kakogawa, Japan

[*]**Corresponding author**: Tomoharu Yokooji, PhD
Department of Pharmaceutical Services, Graduate School of Biomedical and Health Sciences, Hiroshima University, 1-2-3 Kasumi, Minami-ku, Hiroshima 734-8553, Japan.
Tel: +81-82-257-5295, E-mail: yokooji@hiroshima-u.ac.jp

Western blotting

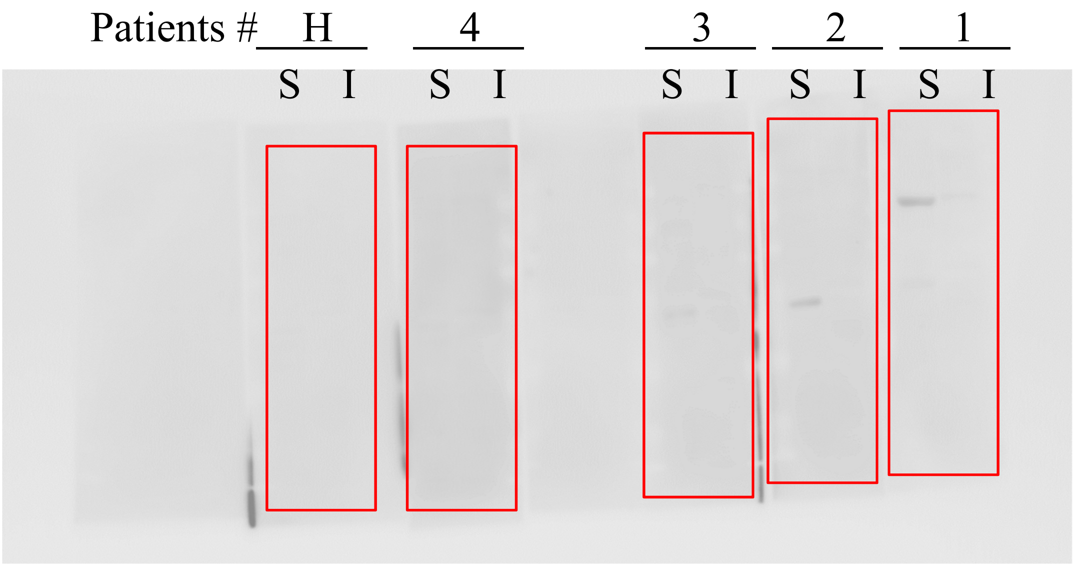

CBB staining

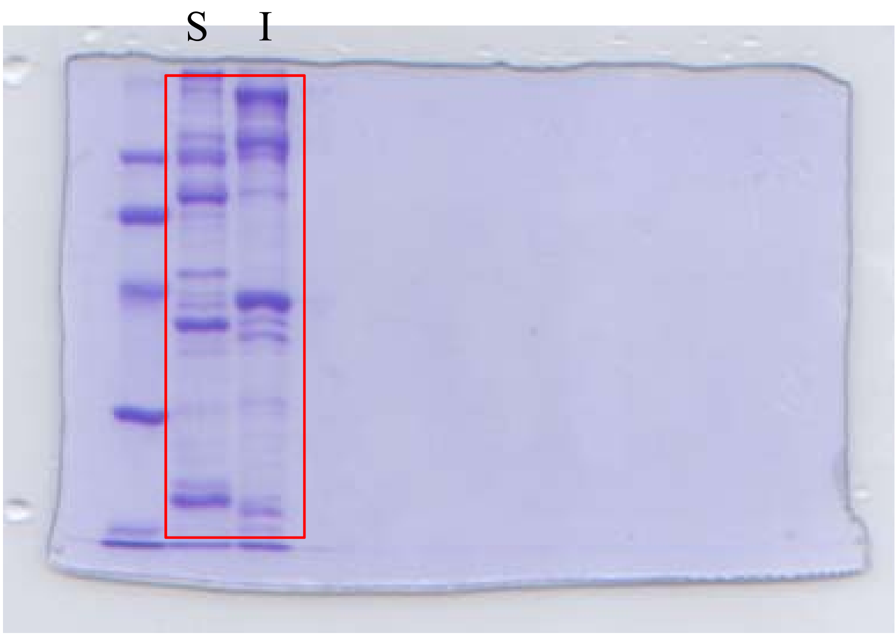

S: Tris-soluble fraction
I: Tris-insoluble fraction
H: healthy subject

Supplementary Figure S1. Uncropped images of Figure 1.
Red boxes denote the areas shown in Figure 1.

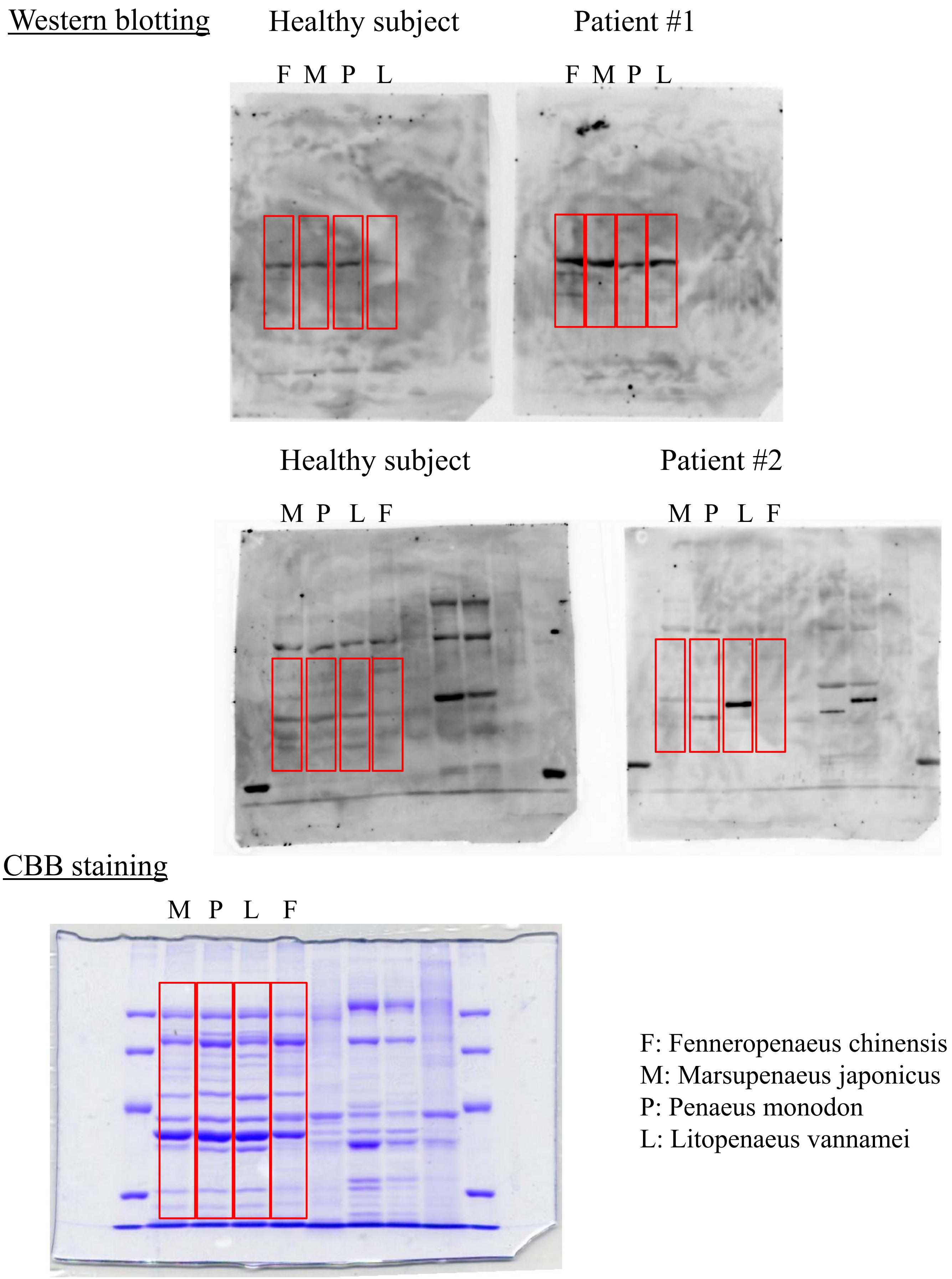

Supplementary Figure S2. Uncropped images of Figure 2.

Red boxes denote the areas shown in Figure 2.

Western blotting

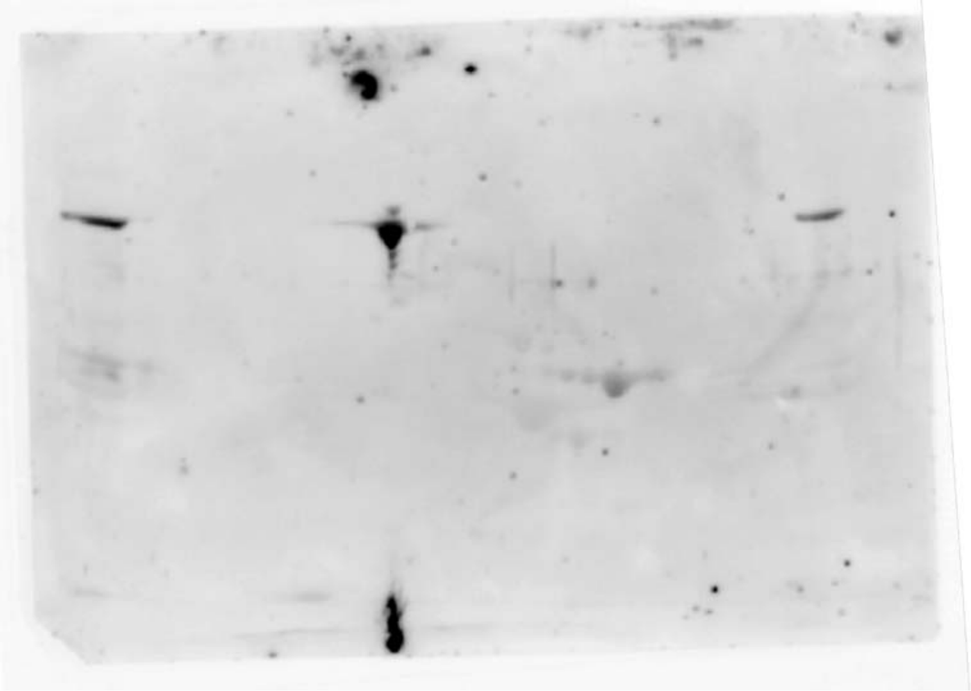

CBB staining

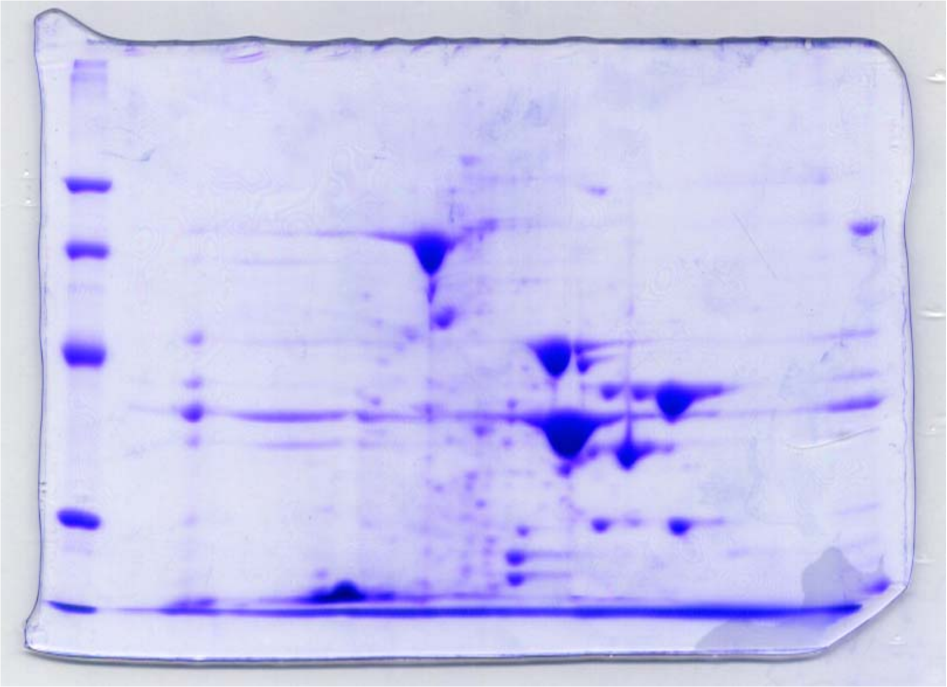

Supplementary Figure S3. Uncropped images of Figure 3.

a 70-kDa protein

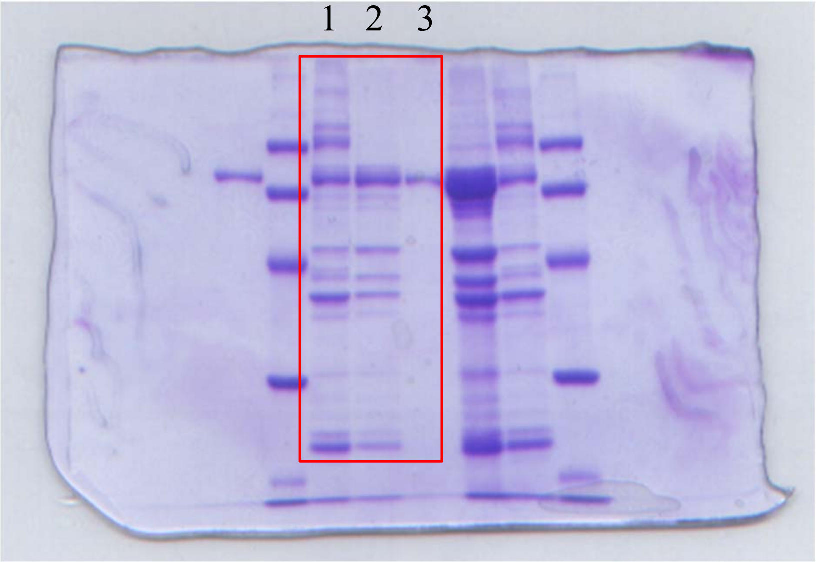

1: whole Tris-soluble fraction
2: the 40–60% ammonium sulfate precipitate
3: reversed-phase HPLC fraction

a 43-kDa protein

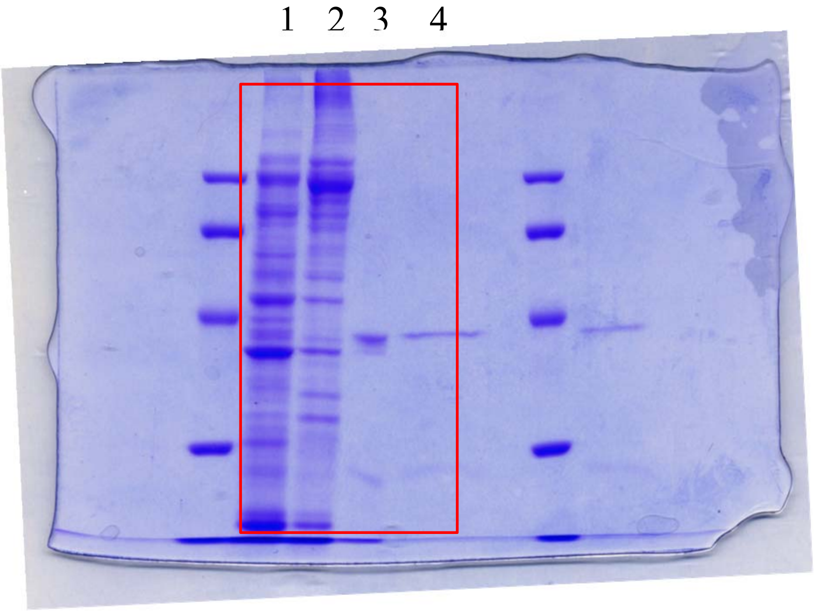

1: whole Tris-soluble fraction
2: the 20–40% ammonium sulfate precipitate
3: anion-exchange chromatography fraction
4: hydrophobic interaction chromatography fraction

Supplementary Figure S4. Uncropped images of Figure 4.
Red boxes denote the areas shown in Figure 4.

P75 homologue

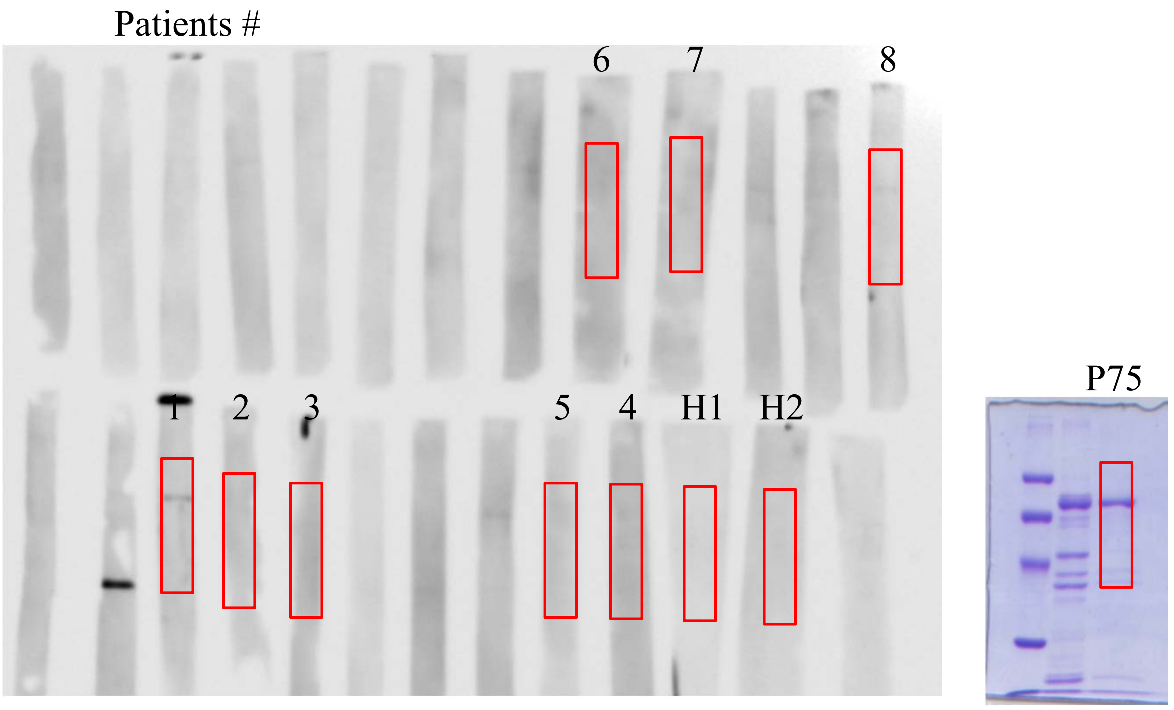

FBPA

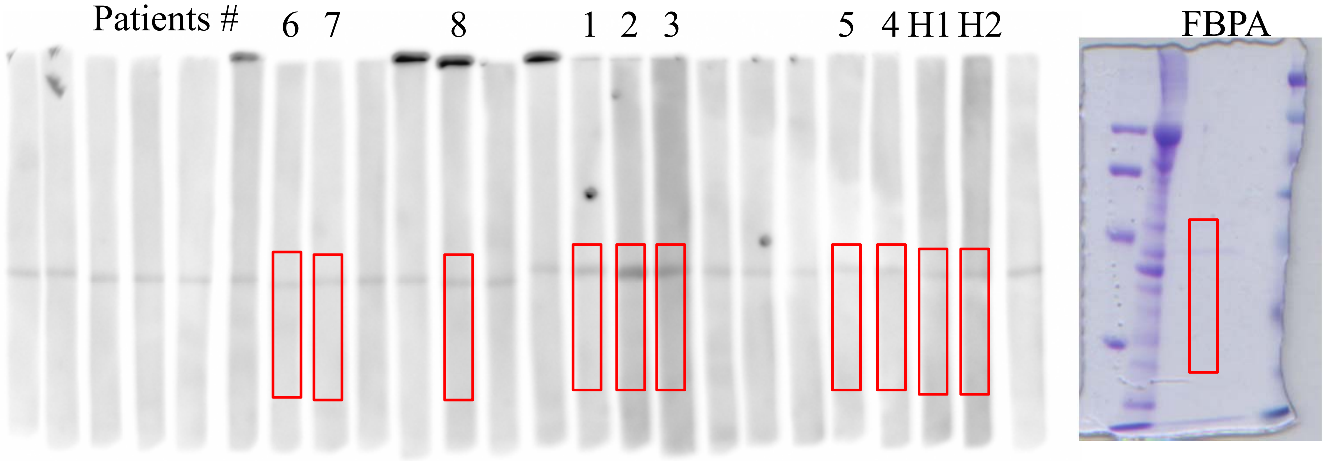

H: healthy subjects

P75: purified native P75 homologue

FBPA: purified native FBPA

Supplementary Figure S5. Uncropped images of Figure 5.
Red boxes denote the areas shown in Figure 5.
